# Incidence and Complications of Traditional Eye Medications in Nigeria in a Teaching Hospital

**DOI:** 10.4103/0974-9233.71596

**Published:** 2010

**Authors:** Catherine U. Ukponmwan, Nanaiashat Momoh

**Affiliations:** Department of Ophthalmology, University of Benin Teaching Hospital, Benin City, Nigeria

**Keywords:** Complications, Cornea, Endophthalmitis, Enucleation, Panophthalmitis, Traditional Eye Medications

## Abstract

**Purpose:**

The aim of this study was to determine the types and nature of traditional eye medications (TEMs), their sources, and the ocular complications that may arise from use in a teaching hospital in Nigeria.

**Materials and Methods:**

A prospective study of consecutive subjects who used TEM before presentation to the Eye Clinic of the University of Benin Teaching Hospital, Benin City, Nigeria between July 1, 2004 and June 30, 2008. *P* < 0.05 was considered statistically significant.

**Results:**

A total of 113 subjects were evaluated of which 64 were males (56.6%), females (43.4%) were females. There was no significant difference in the number of males and females (*P* > 0.05). Rural dwellers were more likely to use TEM than urban dwellers (*P* < 0.0001). The mean age of the subjects was 47.9 ± 22.3 years (range, 4-90 years). The most common traditional medication was derived from plant extracts (54.9%) followed by concoctions (21.2%). Complications occurred in 54.8% of the subjects. Ocular complications included corneal opacities in 13.35% of subjects, staphyloma in 9%, and corneal ulcers in 8%. Other complications were panophthalmitis, endophthalmitis, uveitis, cataract, and bullous keratopathy. Eleven subjects underwent evisceration or enucleation of the affected eye. There was no significant difference in the type of medication used and ocular complications (*P* = 0.956). Sources of TEM were self-medication in 38.9% of subjects, relatives in 27.4%, and traditional healers in 17.7%.

**Conclusion:**

The use of TEM is a common practice that could be harmful and lead to blindness. Proper health education of the public and traditional healers can reduce the prevalence of preventable blindness.

## INTRODUCTION

The use of traditional eye medications (TEM) is still a common practice, as most patients in Africa consult a traditional healer before presentation to a hospital.^1^–^6^ This is despite the well-documented toxic effects of TEM.

Previous studies have reported poverty, poor health seeking behavior, socio-cultural beliefs, and lack of access to health facilities as common reasons for the persistence of this practice.^7^,^8^ The increasing worldwide interest in the use of herbal medicines could also be a factor.^3^,^9^

Practitioners are recognized as traditional medical doctors and although some of their medications are certified as safe by the national drug-regulating agency, they are not meant for ophthalmic use.

Traditional healers tend to prefer the use of substances that cause irritation and pain as this is perceived by the healers and patients as more potent. Such substances may be acidic or alkaline resulting in ocular burns. No particular attention is paid to the mode of action (antibiotic or steroid), concentration, and sterility as most of these concoctions (mixture of various substances which may be plant or animal extracts) are made without regard for hygiene including using contaminated water, local gin, saliva, and even urine.^1^,^4^

Self-medication is a factor that has to be considered as large numbers of patients use plant extracts or concoctions that they make themselves, or are supplied by neighbors and relatives. The perception of supernatural forces as the cause of blindness has also been documented as a barrier to the use of orthodox medications.^1^,^6^,^7^

Various studies in Africa have reported that a large number of patients still use TEM before presentation to the hospital^1^–^4^,^10^,^11^ Courtright *et al*.^10^ reported that 33.8% of patients with corneal disease in Malawi used TEM before presentation to a hospital. Mselle^5^ reported the use of TEM in 49% of Tanzania patients with eye injury. A previous study in Benin, Nigeria reported that 1.72% of patients seen at a hospital-based eye clinic over a 6-month period had ocular complications from the use of TEM.^1^

The objective of this prospective study was to determine the incidence of the use of TEM, the types used and their sources among patients attending the eye clinic of the University of Benin Teaching Hospital, Benin City, Nigeria. The outcomes of this study can aid in determining the ocular complications that may arise from the use of TEM and suggest methods to prevent visual morbidity from these complications.

## MATERIALS AND METHODS

This was a prospective study that comprised consecutive subjects who used TEM prior to presentation at the eye clinic of the University of Benin Teaching Hospital, Nigeria between July 1,2004 and June 30, 2008. All applicable institutional and government regulations concerning the ethical use of human volunteers were followed during this research. Approval for this study was granted by the Ethics Committee of the University of Benin Teaching Hospital. An interviewer administered questionnaire was used during the study. All new patients seen in the eye clinic during the study period were asked about the use of TEM. Only those who used TEM were included in this study. Some of the subjects required hospitalization upon initial presentation due to complications such as hypopyon, corneal ulcers, endophthalmitis, and panophthalmitis.

Patients’ demographics such as sex, age, occupation, and whether the subjects lived in a rural area or in the city were recorded. The symptoms necessitating the use of TEM, the type and source of TEM, history of trauma preceding use of TEM, complications, surgeries, presenting visual acuity, and final visual outcome were documented. The visual acuity was recorded in the Snellen’s notatation.

A penlight exam was performed followed by flourescein stain with slit lamp biomicroscopy to detect corneal lesions. Dilated fundoscopy was performed with the Welch Allyn specialist ophthalmoscope (Welch Allyn Inc., Skaneateles Falls, NY, USA) after instillation of 0.5% tropicamide drops. The intraocular pressure was measured with a noncontact tonometer. Retinal and corneal photography was performed on some subjects for documentation purposes. Photographs showing some of the complications were also taken.

Data analysis was performed with SPSS 13 (SPSS Inc. Chicago, IL, USA) including calculation of frequency tables, mean, mode, and Chi-square test to determine the statistical significance of variables. The statistically significant level (*P*) was set at 0.05.

## RESULTS

**Figure 1:**
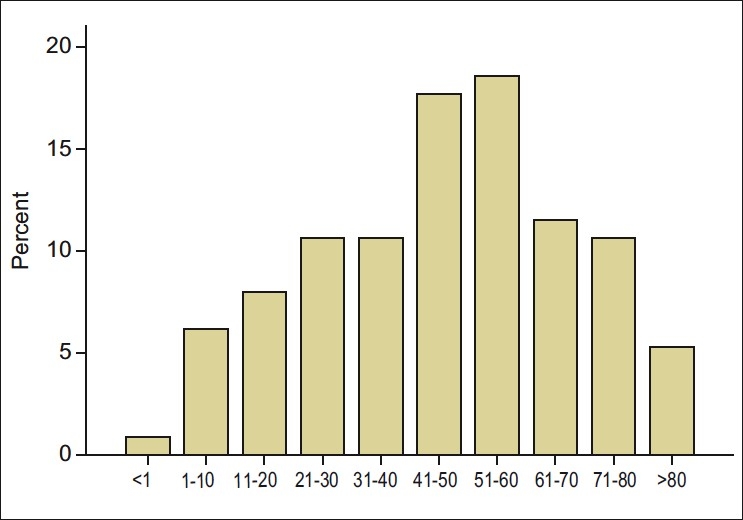
The age distribution of patients

A total of 7220 new patients were seen at the eye clinic during the study period, and 113 (1.57%) of these patients had used TEM before presentation. The study cohort comprised 64 (56.6%) males and 49 (43.4%) females. There was no statistical difference in the distribution of males and females (*P* = 0.1592). Figure 1 shows the age distribution of patients who used TEM. The mean age of the cohort was 47.8 ± 22.3 years (range, 4 months to 90 years). Sixty-two (54.9%) of the subjects were above 40 years. The highest number of subjects (20, 17.7%) was between 51 and 60 years. There was no significant difference between the age and sex of the patients (*P* = 0.320).Sixty-two (54.9%) of these patients resided in rural areas. The rural dwellers were more likely to use TEM compared to urban dwellers (*P* < 0.0001). There was no significant difference between the ages of the subjects and urban or rural residence (*P* > 0.05). During this study, 6903 of 7220 (95.6%) new patients were urban residents and 307 (4.4%) patients were rural residents.

Table 1presents the occupation of subjects, who used TEM. Farmers constituted the highest occupational group accounting for 30.1%, whereas native doctors constituted the lowest group (1.8%) [Table 1].

**Table 1:** The occupation of the patients

Occupation	Frequency	Percentage
Farmer	34	30.1
Artisan	19	16.8
Trader	15	13.3
Unemployed	12	10.6
Civil servant	9	8.0
Driver	9	8.0
Student	8	7.1
Pre-school	5	4.4
Native doctor	2	1.8

Table 2 documents the symptoms leading to the use of TEM, which included pain, redness, discharge, itching, poor vision, trauma, and white spot. The most common symptom was poor vision in 57 patients (50.4%) followed by inflammatory symptoms (redness, pain, and itching) in 43 patients (38.1%) [Table 2]. There was a significant difference between the age of the subjects and the symptoms leading to the use of TEM (*P* = 0.00). There was a significant difference between the occupation of the subjects and the symptoms necessitating the use of TEM (*P* = 0.002).

**Table 2:** The symptoms necessitating the use of traditional eye medications

Symptoms	Frequency	Percent
Poor vision	57	50.4
Inflammatory symptoms	43	38.1
Trauma	7	6.2
White spot	2	1.8
Facial rash	2	1.8
Jaundice	1	0.9
Foreign body	1	0.9

Table 3 presents common TEM used by the study cohort. The most common TEM was plant extract by 62(54.9%) subjects [Table 3]. Any part of the plant may be used including the leaves, flowers or fruits, the stem and the roots. The plant may be ground and used as a paste on the eye or diluted with water or any other liquid such as breast milk, alcohol, and used as an eye drop. The leaves, stem or root of the plant is sometimes dried, ground and mixed with other herbs or liquid and then applied to the eye.

**Table 3:** The types of traditional eye medications used

Types	Frequency	Percent
Plant extract	62	54.9
Concoctions	24	21.2
Breastmilk	4	3.5
Religious items	4	3.5
Lead antimony	2	1.8
Urine	2	1.8
Soot	1	0.9
Alcohol	1	0.9
Not known	13	11.5

Sources of TEM were: 38.9% of the subjects made these concoctions themselves, 27.45% received TEM from relatives, 17.7% consulted a traditional healer, 13.27% received TEM from neighbors; and in 2.65% of subjects the source was unspecified. There was no significant difference between the age of the patients and the source of TEM (*P* > 0.5).

**Figure 2:**
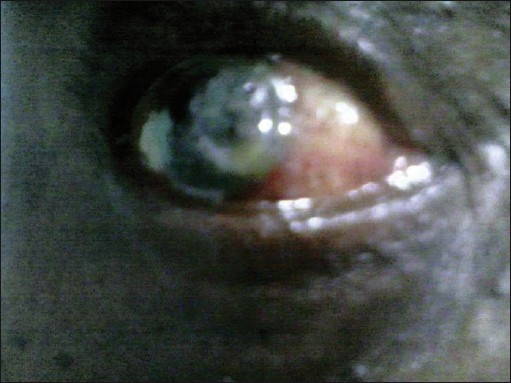
Eye with panophthalmitis secondary to traditional eye medication

Ocular complications occurred in 54.8% of the cohort [Table 4]. The most common complications were corneal opacity in 17 subjects [Table 4]. One subject had bilateral corneal opacities, and hypopyon was present in 19 eyes [Table 4]. Thus, complications involving the cornea including staphyloma occurred in 51 (45%) subjects. Six subjects (5.3%) underwent evisceration, five (4.5%) subjects underwent enucleation of the affected eye. There was no significant difference between the type of TEM and the development of ocular complications (*P* =0.956). Figure 2 shows the eye of a subject who developed panophthalmitis from the use of TEM.

**Table 4:** Complications

Complications	Frequency	Percent
Corneal opacity	17	15.0
Staphyloma	13	11.5
Pan/endophthalmitis	7	6.2
Corneal perforation	7	6.2
Corneal ulcer	6	5.3
Band keratopathy	5	4.4
Uveitis	3	2.7
Bullous keratopathy	2	1.8
Subluxated lens	1	0.9
Complicated cataract	1	0.9
Descemetocoele	1	0.9
None	50	44.2

**Figure 3:**
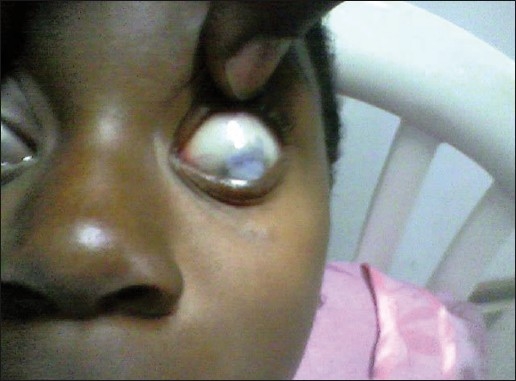
Eye with corneal opacity secondary to traditional eye medication

Figure 3presents an eye with corneal opacity due to TEM. There was no significant difference between the age of the subjects and the development of ocular complications (*P* = 0.363).

**Figure 4:**
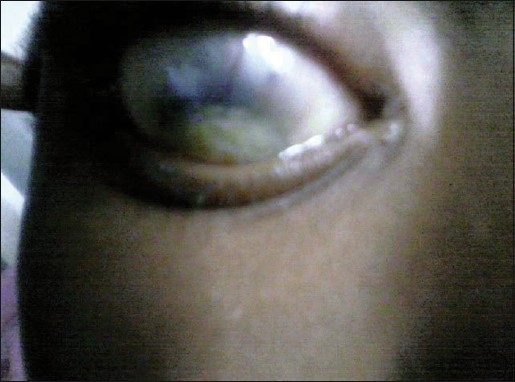
Eye with corneal opacity secondary to traditional eye medication

The visual acuity improved in 5.3% of the subjects, there was no improvement in 83.2% of the subjects and 11.5% had reduction in visual acuity after treatment. Ninety-two subjects were blind in the affected eye with a final visual acuity of less than 3/60 as shown in Table 5. One subject was blind bilaterally from corneal opacities due to the use of TEM. Figure 4 shows an eye with a corneal opacity from the use of TEM. Some of these subjects were already blind or had poor vision from pre-existing ocular diseases such as cataract and glaucoma and this precipitated the use of TEM.

**Table 5:** The visual acuity of the patients

Visual acuity	Number of patients	Presenting VA (%)	Number of patients	Final VA (%)
6/5-6/18	8	7.1	10	8.8
<6/18-6/60	2	1.8	3	2.7
<6/60-3/60	6	5.3	8	7.1
<3/60-LP	74	65.5	63	55.8
NLP	23	20.4	29	25.7

## DISCUSSION

The results of this study confirm previous findings that the use of TEM can further degrade visual outcomes in patients with minor ocular problems.^1^–^5^,^9^ More than half the total number of all the new patients in this study were 40 years or older, and it is, therefore, not surprising that 54.9% of the subjects that used TEM were above 40 years.

In Nigeria, a large proportion of the population resides in rural areas where farming is a major occupation. There are also other outdoor workers such as traders and artisans. These workers are exposed to trauma and a climate that predispose them to ocular conditions such as abrasions, lacerations, allergic, and bacterial conjunctivitis which is compounded by poverty and lack of access to medical care. Such factors are an impetus to use of TEM. In this study, 95.6% of all new patients seen in the eye clinic were living in urban areas, whereas 54.9% of the subjects who used TEM lived in rural areas. Rural residence was found to be an important factor in the use of TEM in this study, and there was a statistically significant difference between urban or rural residence and the use of TEM (*P* < 0.0001). The lack of access to hospitals, due to proximity and relatively access to TEM through friends, relatives, and neighbors likely explains the preponderance of rural subjects resorting to TEM.

Beliefs regarding the cause of diseases may also play an important role because many people in rural areas believe that diseases are caused by breaking taboos or not conforming to traditional societal rules and hence they tend to consult a traditional healer or elders of the community in the event of an ocular disease. The inability of patients to afford the high cost of services in the hospitals is also another reason given by some patients for their use of TEM before presentation to the hospital. This is shown, in this study, where only 7220 new patients were seen in the eye clinic during the study period. The low number of patients was due to the reluctance of patients to take up eye care services in the hospital due to barriers such as lack of accessibility and high cost of services. This shows that high cost of services, distance from the hospital, and nonavailability of an eye center near the place of residence may contribute to the use of TEM.

A report from Oman found that 54.8% of the rural population used traditional medication before presentation to the hospital.^9^

The symptoms necessitating the use of toxic medications as seen in this study ranged from minor symptoms of inflammation (probably due to conjunctivitis, episcleritis, scleritis, and uveitis), poor vision (which could have been due to refractive errors or cataract), trauma, and whitish spot (due to congenital or developmental cataract) which could easily have been handled by an ophthalmologist resulting in good visual prognosis. There was a significant difference between the age of the patients and the symptoms responsible for the use of TEM (*P* = 0.00). The use of TEM is widespread in Nigeria, yet there is a dearth of literature on the prevalence.^1^ There is a need to study the overall prevalence of the use of TEM in the population. This will aid in assessing the risk of TEM use in the population.

Various studies from Africa and Asia have reported the large-scale use of TEM for corneal ulcers, ocular injuries, and other eye diseases.^1^,^3^,^10^–^15^ Courtright *et al*.^10^ reported that 33.8% of patients with corneal ulcers in rural Malawi used TEM before presentation to hospital. Prajna *et al*.,^11^ reported that 47.7% of patients with corneal ulcers in South India and Singh^12^ from Nepal reported that 57% of the patients with corneal ulcers used TEM. In Tanzania, 49% of patients with ocular injuries used TEM.^3^ An earlier study of corneal ulcers in Benin also reported the use of TEM by many of the patients.^13^ The use of harmful TEM has been reported in epidemics of acute hemorrhagic conjunctivitis in Africa.^14^ A study by Yorston and Forster^15^ in Tanzania revealed that 25% of corneal ulcers in 103 patients were associated with the use of TEM within the previous 7 days.

The use of TEM by 54.9% of subjects could be due to the fact that farmers residing in rural areas constituted the highest occupational group. A large proportion of subjects presented when they were already blind (VA < 3/60 to no light perception) by complications of TEM. In this study, 54.8% of subjects who used TEM developed complications. Corneal opacities, staphyloma, and corneal ulcers were the most common complications, and these could have resulted from the alkaline or acidic contents of the concoctions which are toxic to the cornea. Most of these traditional medications are often prepared with alkaline solutions and not acidic solutions, hence the high degree of corneal damage and scarring. TEM may also cause corneal damage by introducing micro-organisms into the eye, which lead to primary or secondary infection. Ocular complications such as keratitis, endophthalmitis, and panophthalmitis were more frequent in patients with a positive history of TEM than those with a negative TEM history in Tanzania.^5^ The secondary infections are likely to be due to the unsanitary condition used to make TEM.

The poor visual outcome from the use of TEM was further buttressed by the finding that visual acuity remained unchanged after treatment in 83.2% of subjects due to reduction or loss of vision from corneal opacity and staphyloma. Panophthalmitis, endophthalmitis, or subsequent removal of the involved eye caused reduced vision in 11.5% of the subjects. Only 5.31% of the subjects had improved vision after treatment. Bilateral blindness from complication of TEM was seen in 5 of 30 patients in an earlier report from the same hospital.^1^ Lewallan *et al*.^16^ reported that peripheral corneal ulcers were associated with the use of TEM in their study. Chirambo *et al*.^17^ found out that 26% of blindness among blind school children was from TEM use.

This study has shown that many patients still use TEM for various ocular conditions including conjunctivitis, corneal ulcers, and trauma, which can cause blindness due to associated complications. Most of these ocular conditions can be readily addressed by orthodox medicine which would circumvent the major cause of visual morbidity associated with TEM. The prevalence of this preventable blindness can be reduced by intensive health education to the communities, both urban and rural, to dissuade them from self-medication and consultation with untrained personnel. Clearly, the population should be educated about the dangers of TEM. There is no existing partnership between traditional healers and the prevention of blindness programmes in Nigeria. However, such a partnership is required to educate traditional healers about the importance of referring patients with ocular complaints to the hospital. There is a need to establish trust and respect among healthcare providers, patients, and the communities.

Primary eye care workers have a very important role to play in the prevention of blindness from TEM. They are often the first point of contact when a complication occurs with treatment and their contact with the community is important in discouraging TEM use. There is a need to send healthcare personnel to community health centers, and to improve and upgrade primary eye care programmes in Nigeria. Nurses and community health extension workers should be trained to recognize and promptly refer cases of corneal ulcers and trauma to ophthalmologists. Publicity of unsubstantiated claims by traditional medicine practitioners of the efficacy of herbal extracts and concoctions should be strongly discouraged.
